# The influence of exotic and native plants on illnesses with physical and spiritual causes in the semiarid region of Piauí, Northeast of Brazil

**DOI:** 10.1186/s13002-024-00667-y

**Published:** 2024-02-26

**Authors:** Paulo Henrique da Silva, Washington Soares Ferreira Júnior, Sofia Zank, André Luiz Borba do Nascimento, Maria Carolina de Abreu

**Affiliations:** 1 Programa de Pós-Graduação em Biodiversidade e Conservação (PPGBC), Universidade Federal do Piauí (UFPI), Floriano, Piauí Brazil; 2 Laboratório de Estudos Etnobiológicos (LEET), Universidade de Pernambuco (UPE), Nazaré da Mata, Pernambuco Brazil; 3 Laboratório de Ecologia Humana e Etnobotânica (ECOHE), Universidade Federal de Santa Catarina (UFSC), Florianópolis, Brazil; 4 Programa de Pós-Graduação em Biodiversidade e Conservação (PPGBC), Universidade Federal do Maranhão (UFMA), São Luís, Maranhão Brazil

**Keywords:** Caatinga, Ethnobiology, Diversification hypothesis, Utilitarian redundancy, Therapeutic versatility

## Abstract

**Background:**

Local medical systems (LMS) include native and exotic plants used for the treatment of diseases of physical and spiritual nature. The incorporation of exotic plants into these systems has been the subject of many studies. In this context, an analysis was conducted on the influence of the origin of plants on diseases of physical and spiritual nature in order to evaluate the therapeutic versatility of native and exotic species in these therapeutic targets, to investigate whether exotic plants mainly fill gaps not met by native plants (diversification hypothesis), and identify which species are prioritized in the redundant targets in these two therapeutic groups in the rural community of Morrinhos, Monsenhor Hipólito, Piauí.

**Methods:**

Data collection took place in 2 stages. First, free lists and semi-structured interviews with local residents (*n* = 134) were conducted to survey plants used for therapeutic purposes and the associated illnesses. Then, another phase of interviews was carried out to evaluate the prioritization between native and exotic plants in redundant therapeutic targets. To test the diversification hypothesis (DH) in each group of illnesses, data were analyzed using generalized linear models (Poisson and Binomial GLMs); versatility was measured by the number of therapeutic indications and compared between resources using the Mann–Whitney test, and prioritization in each group was verified by comparing the proportions of native and exotic plants with the χ^2^ test.

**Results:**

One hundred and thirty-two species of plants were surveyed, being 71 exotic and 61 native, with indications for physical and spiritual illnesses. The results revealed that the diversification hypothesis did not explain the inclusion of exotic plants in the local medical system to treat physical or spiritual illnesses and that the therapeutic versatility of exotic and native resources in the two groups was also similar (*p* > 0.05). However, exotic plants were prioritized in illnesses with physical causes and native plants in illnesses with spiritual causes.

**Conclusions:**

The local medical system presents similar and distinct patterns in the therapeutic targets, depending on the perspective evaluated. Therefore, it is necessary to investigate the patterns of use of medicinal plants in different sociocultural contexts in order to broaden the debate about the role of plant origin in the selection of treatments for illnesses with different causes.

## Background

Through the therapeutic use of plants, human groups have developed classification systems, beliefs and practices that are important for the treatment of the illnesses that affect them [1]. Knowledge systems that encompass the use of medicinal natural resources are known as local medical systems (LMS) and involve the active participation of individuals in diagnosis, therapy, and healing strategies [2]. These medical systems are composed of both native species that occur in the natural areas where a particular human group is settled and exotic species originating from other regions, countries or continents [3]. The incorporation of exotic species in LMS has been the investigative scope of different studies over time [4–9].

Some researchers have observed that one of the aspects that contribute to the incorporation of exotic plants in LMS is their versatility in relation to native plants. It is likely that these plants are firstly adopted for purposes other than medicinal applications, such as food or ornamental use, and it is only after continuous experience and use that they become part of the medicinal repertoire, as suggested by the versatility hypothesis [10]. In this case, the more versatile an exotic species is, the more likely it is to be incorporated into the daily lives of local populations [11]. However, no evidence supporting this idea was found by Alencar et al. [6] in a study conducted in the Caatinga, in Northeastern Brazil, since native plants showed greater versatility in terms of general uses. Furthermore, the authors found no significant differences between native and exotic plants with regard to medicinal versatility, demonstrating the need to investigate more closely the factors that determine the inclusion of exotic species in LMS. In view of this, it has been suggested that once inserted in LMS, exotic species offer two possibilities of action: (1) they expand the capacity of the medical system to resolve health problems, treating therapeutic targets to which no medicinal resource is found among native species; or (2) they provide new therapeutic options for diseases already treated with native species, favoring utilitarian redundancy [7].

The idea of the first possibility was raised in the work of Albuquerque [4], who proposed the diversification hypothesis (DH), which sustains that exotic plants are introduced in LMS to fill therapeutic gaps left by native species, thus diversifying the therapeutic spectrum of the system. Some studies have already tested this hypothesis, but evidence supporting it is still inconclusive [6, 9, 12, 13]. As for the second possibility, we first need to understand the concept of utilitarian redundancy. The term was introduced in the work of Albuquerque and Oliveira [5] who, based on an analogous concept (ecological redundancy), developed the utilitarian redundancy model (URM) with the purpose of evaluating the functional overlap of resources in LMS. The use of several medicinal species with the same therapeutic function indicates the existence of redundancy in this function within the system [14]. Consequently, the increase of utilitarian redundancy in a system implies a growth in the variety of treatment options, providing flexibility of choice for people [15]. This flexibility is a crucial component of the resilience of the system [15], that is, its ability to absorb disturbances and adapt to environmental changes, maintaining therapeutic functions and processes [16, 17].

The presence of exotic plants in LMS can also reduce the use-pressure on redundant native species in the same function [18]. This is because the pressure is shared with the exotic species, and the latter can even be used more often than native plants that perform the same function in the system [19]. Although the use of exotic plants in the systems may reflect an acculturation process in the view of some authors, it may actually involve choices based on adaptive advantages, influencing human behavior toward the management of these plant resources [15, 20].

The LMS and healing practices around the world show that each cultural group has a unique understanding of illnesses and of how they connect with cosmological spheres, which is reflected on therapeutic aspects [21]. Studies have indicated that the cause of the illness influences the treatment, leading to different strategies in healing systems, depending on the perceived context [21–23]. From this perspective, the causes of illnesses or “therapeutic targets” may present variations depending on the perception of human groups. They can be categorized into: (1) illnesses or therapeutic targets with physical causes, when they are related to natural causes and linked to physical perceptions, with some degree of correspondence with biomedical systems [21, 24, 25]; or (2) illnesses or therapeutic targets with spiritual causes, when the causes are not recognized by biomedicine, but are associated with a symbolic and immaterial dimension, with explanations linked to a supernatural atmosphere accepted by a human group or ethnicity [21, 26, 27]. Thus, disease and health are not limited to biological and objective configurations, but are rather sociocultural constructions that vary according to each social group and its cultural identity [28, 29].

Plants play a fundamental role within the sociocultural context of health and disease. They are used to treat not only diseases caused by physical factors, but also disorders of a spiritual nature; they are used for example for energy cleansing, “mau olhado” (evil eye), “vento-caído” (‘fallen wind’, when a child takes a fright), “quebranto” (‘brokenness’), spells, and so on [26, 30, 31]. Exotic plants have been incorporated into spiritual therapeutic practices, including rituals and related therapies, resulting in modifications within cultural systems [32]. However, the role of the perceived causes of spiritual diseases in the process of incorporation of exotic species into LMS has been addressed only in a few studies.

In these circumstances, it is essential to investigate the patterns of use of plant resources [6] in therapeutic targets involving different causes (physical and spiritual) because, in LMS, the perceived cause of the illness is usually the factor that triggers the treatment [21]. This approach will allow a more detailed understanding of the dynamics of these resources in the local system, since previous studies investigating the incorporation of plants aimed at therapeutic targets addressed the medical system in general, encompassing a variety of diseases without performing a specific analysis of their perceived causes.

In the present study, we propose the rural community of Morrinhos, an area of Caatinga in the municipality of Monsenhor Hipólito, state of Piauí, Brazil, as scenario for the study of the following central question: what is the influence of the origin of plants (native and exotic) in the selection of treatments for illnesses with different perceived causes (physical and spiritual) in the LMS? Through the survey of the plants used in therapeutic practices in this community, this study primarily aims: (a) to investigate whether exotic species predominantly fill therapeutic gaps left by native species in illnesses with physical and spiritual causes (diversification hypothesis); (b) to evaluate the versatility of native and exotic species in these therapeutic targets separately; and (c) to analyze the utilitarian redundancy between native and exotic species in these therapeutic targets, identifying the species prioritized in each redundant target.

## Material and methods

### Study area

**Fig. 1:**
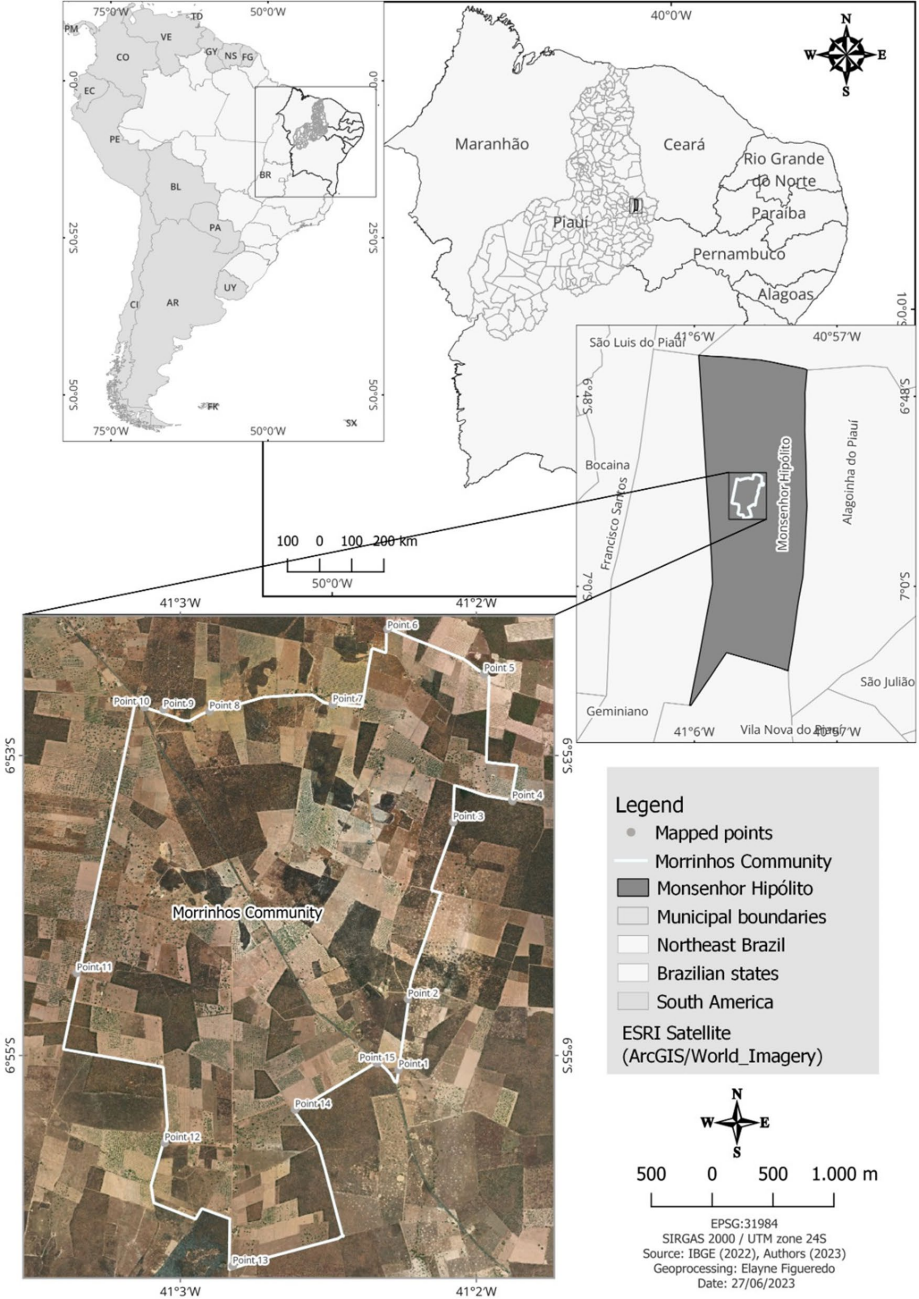
Location of the rural community of Morrinhos, Monsenhor Hipólito, Piauí, Northeastern Brazil

This is an ethnobotanical study with a qualitative and quantitative approach conducted in the rural community of Morrinhos, located in the municipality of Monsenhor Hipólito, Piauí, Northeastern Brazil, about 14 km from the headquarters of the municipality (Fig. 1). The municipality has a territory of 401.568 km^2^ (06º59′47″ S and 41°01′47″ W) and a population of 7577 inhabitants, of whom more than half live in rural areas [33]. Regarding physiographic aspects, the area has a hot semiarid tropical climate [34] and mixed vegetation of a transition zone between Caatinga and Cerrado (deciduous/hyperxerophyllous Caatinga forest and sub-deciduous Cerrado) [35]. The soils of the region are derived from the alteration of sandstones, shale, conglomerate and siltstone, comprising litholic, alic, dystrophic soils in association with red-yellow podzol soils and quartz sands [35].

According to information provided by the two community health agents working in the area, obtained through the Family Health Program (FHP), the community is organized into family groups and has about 340 residents distributed in 120 households. The residents basically survive on subsistence agriculture, with seasonal cultivation of beans, corn and cassava, in addition to cashew harvesting as main activities. Rearing of cattle and small animals such as chickens, guineas and pigs is also practiced. The production is consumed within the community and the surplus is sold at the municipality’s open market or sold to local or regional buyers, generating income and businesses.

There are some public services in the region, such as three Primary schools (one school for early childhood education and two schools for elementary education), a health center serving local residents and the surrounding communities, and public school transport to take students to the urban area for them to attend school grades not available in the community. The community has access to electricity, piped water (most of the population), media, and internet. The area has an easy access, with good quality side roads, and it is crossed by the Benedito Joaquim de Carvalho highway (PI-229), which guarantees good mobility and production flow.

In terms of religions, Catholicism predominates. For health care, in addition to biomedicine and industrialized medicines, the residents use plant resources grown in backyards/farmyards, extracted from natural areas or acquired at fairs and markets, in order to complement the therapeutic system and seek their well-being. Many have a clear preference for these resources over biomedicine. Ritualistic practices of blessings to treat problems that cause affliction, as well as the use of protective amulets, are also common.

### Ethical and legal aspects

This study was approved by the Ethics and Research Committee of the Federal University of Piauí through the Brazil Platform under opinion number 5637518 (CAAE: 60898722.3.0000.8057), as provided for in Resolutions 466/2012 and 510/2016 of the National Health Council, which regulate research involving human beings. Each participant signed an Informed Consent Form (ICF) and was informed of the guarantee of confidentiality of the information collected, integrity, and the right to withdraw at any time. The study was registered in the National System of Genetic Resource Management and Associated Traditional Knowledge (SisGen) under number AADE637 and received authorization from SISBIO (Biodiversity Information and Authorization System) for collection of botanical material (registration number 85297-1).

### Data collection

The community health agents were initially contacted and invited to provide information about the community in general and the residents assisted by them and then the guidelines and stages of the study were presented to them. After that, the objectives of the study were presented to some selected community leaders (religious leaders, health professionals, rural unionists). After this phase of clarifications, data collection was implemented in two stages, in the period between November/2022 and May/2023.

A stratified representative sample was taken from a population composed of residents aged 18 years or older (*n* = 205 adults), following a 95% confidence level, resulting in a sample of 134 people. The community is structured in nuclei: Morrinhos I, II, III and IV. The sampling was performed proportionally and randomly to represent these nuclei, corresponding, respectively, to 22% (29), 22.4% (30), 17.1% (23) and 38.5% (52) of the sample set.

In the first stage, visits were made to the residents of the community. The technique of free listing of plants used for therapeutic purposes was initially applied to each participant, through the question “What plants do you know to treat illnesses with physical and spiritual causes?” associated with nonspecific induction and the technique of re-reading at the end of the list. After listing the plants, an individual semi-structured interview was conducted to explore the mentioned resources and the illnesses associated with them also using the technique of direct observation in the community [36]. The objective of this phase was to gather socioeconomic data and information about the plants used in the treatment of illnesses with physical and spiritual causes based on the relationship of the residents with local plant diversity, covering aspects such as: vernacular names; therapeutic indications; perceived causes of the mentioned targets; plant parts and forms of preparation; places of acquisition, among others. The categorization of the therapeutic targets mentioned according to the perceived causes (physical or spiritual) was made for each illness mentioned by the participant, taking into account the characteristics that they thought to be related: whether the illnesses originated naturally in the body itself or were caused by factors such as microorganisms and/or physicochemical elements, in the case of diseases of physical nature; or if they were attributed to supernatural and energy factors, in the case of spiritual illnesses. During this stage, the expected number of 134 residents were interviewed, being 82 women (61.2%) and 52 men (38.8%), aged between 18 and 85 years.

Parallel to the interviews, the guided tour technique [37] was used to obtain photographic records and collect fertile specimens of the plants mentioned by the participants. The collected material was herborized [38], identified with the aid of analytical keys, specialized bibliographies, and consultations with specialists, and deposited in the collection of the Graziela Barroso Herbarium (TEPB) of the Federal University of Piauí – UFPI as testimony material of this study (voucher). In the case of plant resources acquired at fairs and markets, in which only parts of the plants (whole or processed) were obtained, the most coherent identification, according to the general identification of the products used in the region, was used, because the collection of these plants was not possible.

The classification system adopted was APG IV [39]. The scientific nomenclature of taxa and the status of the plants as native or exotic were checked in the database Flora and Fungi of Brazil (http://floradobrasil.jbrj.gov.br), and when the species were not present in this database, the database of the Missouri Botanical Garden (https:// https://www.tropicos.org/home) was consulted. As for the biogeographic origin of the species, all species with origin in the Brazilian territory were categorized as native, regardless of region or biome, and species with origin outside Brazil were categorized as exotic [6, 8] regardless of the degree of naturalization (see [40]). Also, the term “magical-religious plant” was adopted for plant resources related to therapeutic targets with spiritual causes.

After collecting the data, a second round of interviews was carried out with the same participants of the first interviews to evaluate whether native or exotic species were prioritized for the redundant therapeutic targets mentioned in the first interviews. To this end, the names of the plants mentioned for each redundant target were presented and the participants were asked to order them according to the priority of use [36]. One hundred and thirty-two (132) individuals participated in this stage, and they showed some overlap of species in the therapeutic targets mentioned.

### Data analysis

The collected data were systematized in Microsoft Excel® spreadsheets and descriptive statistics were applied for an overview of the data. Considering the local nosology, the surveyed plants were grouped according to the physical and spiritual therapeutic indications mentioned by the participants [5].

In order to evaluate the versatility of native and exotic species in illnesses of physical and spiritual nature separately in the local medical system, the measure of versatility took into account only the number of therapeutic indications in these 2 groups. The normality of the data in each therapeutic group (physical and spiritual) was verified using the Shapiro–Wilk test (*p* < 0.001 in both). Then, the results between samples of native and exotic species were statistically compared using the nonparametric Mann–Whitney test in order to check if differences between these samples were significant in each of these groups.

To test the diversification hypothesis, the number of native and exotic species mentioned to treat diseases in each therapeutic group was measured. Then, for each group, two generalized linear models were built: a GLM with Poisson distribution and a GLM with binomial distribution. In each model with Poisson distribution, the objective was to investigate whether, in each therapeutic group, exotic resources tended to be fewer when more native resources were available; the richness of exotic species was considered as the dependent variable and the number of native species was the independent variable. In turn, in each Binomial model, the independent variable was the number of native species known to treat each illness, and the dependent variable was the presence (1) or absence (0) of exotic species for this function. The objective was to investigate whether, in each group of illnesses, the therapeutic indications without exotic species presented a greater number of native species.

In order to evaluate whether exotic or native species were prioritized in the treatment of illnesses with physical and spiritual causes, based on the ordering generated by the participants, the first option mentioned in each target was considered and its biogeographic status (native or exotic) was noted. Then, the quantities of prioritized native and exotic species were counted in each therapeutic group and a chi-square test (*χ*^2^) was applied to check if there were differences in the proportions between the targets in which exotic species were more often used *versus* targets in which native species were prioritized.

All analyses were performed in the R statistical environment version 4.2.3 [41]. Therapeutic groups (physical and spiritual) were analyzed separately, and significant results were obtained at *p* < 0.05.

## Results

The 134 participants cited 132 plant species that are known and/or used in the local medical system, distributed in 111 genera and 52 botanical families, with one species identified only at the genus level. Among this set, 71 were exotic (53.8%) and 61 were native to the Brazilian territory (46.2%); among the native species, only 22.9% were endemic to Brazil. Regarding the habit, the exotic species were predominantly herbs (45.1%), followed by trees (25.8%), shrubs (22.5%), lianas (5.6%), and palm trees (1.4%). The native species were mostly trees (44.3%), followed by shrubs (23%), herbs (21.3%) and lianas (11.4%). These plants were indicated for 125 therapeutic targets, 107 of physical nature and 18 of spiritual nature. According to local perception, 106 plants are useful only for therapeutic targets with physical causes, 11 exclusively for therapeutic targets with spiritual causes, and 15 for both types of targets. The exotic species were distributed in 34 families and the native species in 28 families. In general, the botanical families most represented in the LMS in terms of number of species were Fabaceae (16 spp.), Euphorbiaceae (9 ssp.), Lamiaceae and Asteraceae (7 spp. each). The data on plant resources are systematized in Table 1.

**Table 1:** Plant resources and indications for therapeutic targets with physical and spiritual causes in Morrinhos

Family/species	Common name	O	Physical therapeutic targets	Spiritual therapeutic targets	PU	Hb	Vph	Vsp	TEPB
**Amaranthaceae**									
*Alternanthera bettzichiana* (Regel) G.Nicholson	chá-preto	e	Headache, vomiting		lf	H	2	–	32,676
*Alternanthera brasiliana* (L.) Kuntze	anador, penicilina	N	Fever, headache, general pain, sore throat		lf	H	4	–	32,672
*Dysphania ambrosioides* (L.) Mosyakin & Clemants	Mastruz	e	Bone fracture, flu, cough, measles, bone pain, worms, gastritis, inflammations, wounds, back pain, bronchitis, pneumonia, bruises, leg pain, gastric ulcer, stomachache, menstrual cramps, appendicitis, female inflammation, asthma, cancer, headache, healing ointmen, general pain		lf, br	H	24	–	32,559
**Amaryllidaceae**									
*Allium* sp.	cebolinha-branca	e	Cough, colic in babies ("*espremedeira*")		bb	H	2	–	NC
*Allium cepa* L	cebola/onion	e		Ward off evil spirits, protection from negative energies, bad memories	bb	H	–	3	NC
*Allium sativum* L	alho/garlic	e	Flu, cough, gastric ulcer, covid, asthma	Protection from negative energies, envy, brokenness ("*quebranto*"), evil eye	bb	H	5	4	NC
**Anacardiaceae**									
*Anacardium occidentale* L	caju/cashew	n	Healing ointmen, wounds, flu, abdominal pain, diarrhea, stop bleeding, toothache, diabetes, sore throat, urinary tract infection, female inflammation, indigestion		ba, ib, lf	T	12	–	32,560
*Astronium urundeuva* (M. Allemão) Engl	aroeira	n	Inflammations, kidney pain, back pain, uterine inflammation, ovarian inflammation, female inflammation, vaginal discharge, healing ointmen, wounds, diarrhea, urinary tract infection, bone pain, sore throat, flu, prostate problems		ba, ib, lf	T	15	–	32,805
*Mangifera indica* L	manga/mango	e	Flu, indigestion, fever, bone pain, kidney pain		lf	T	5	–	32,561
*Schinopsis brasiliensis* Engl	braúna	n	Abdominal pain, gastritis, wounds, healing ointmen, sinusitis		ba, lf	T	5	–	32,802
*Spondias purpurea* L	seriguela	e	Diarrhea, abdominal pain		lf	T	2	–	32,562
*Spondias tuberosa* Arruda	imbu	n*	Flu		lf	T	1	–	32,563
**Annonaceae**									
*Annona muricata* L	graviola	e	Cancer		lf	T	1	–	32,654
*Annona squamosa* L	ata	e	Abdominal pain, indigestion, healing ointmen		lf, fr	T	3	–	32,564
**Apiaceae**									
*Anethum graveolens* L	endro	e	Indigestion, abdominal pain, constipation (laxative)		sd	T	3	–	32,671
*Coriandrum sativum* L	coentro/cilantro	e	Abdominal pain, indigestion, menstrual cramps, delay aging		sd, lf	T	4	–	32,565
*Foeniculum vulgare* Mill	erva-doce/fennel	e	Tranquilizer, indigestion, abdominal pain, flu, vomiting, insomnia		sd	T	6	–	32,566
**Apocynaceae**									
*Aspidosperma pyrifolium* Mart. & Zucc	pereiro	n	Abortion		lf	T	1	–	32,666
*Calotropis procera* (Aiton) W.T.Aiton	flor-de-seda	e	Cutaneous warts		lt	S	1	–	32,567
**Araceae**									
*Dieffenbachia seguine* (Jacq.) Schott	comigo-ninguém-pode	n		Evil eye, envy	wp	H	–	2	32,695
*Zamioculcas zamiifolia* (G.Lodd.) Engl	amilculca	e		Evil eye	wp	H	–	1	32,703
**Arecaceae**									
*Cocos nucifera* L	coco/coconut	e	Urinary tract infection, diarrhea, abdominal pain, indigestion		fr	PT	4	–	32,655
**Asparagaceae**									
*Aloe vera* (L.) Burm.f	babosa	e	Wounds, flu, cough, expectorant, hair loss, cancer, skin cancer, burn, healing ointmen, inflammations, gastritis, stomachache, worms, constipation (laxative), furuncle, ringworm, female inflammation, dandruff, skin cleansing, hemorrhoid	Evil eye	lf, wp	H	20	1	32,694
*Sansevieria cylindrica* Bojer ex Hook	flecha-de-são-jorge	e		Evil eye, envy	wp	H	–	2	32,697
*Sansevieria trifasciata* Prain	espada-de-são-jorge	e		Evil eye, envy, ward off evil spirits	wp	H	–	3	32,698
**Asteraceae**									
*Acanthospermum hispidum* DC	carrapicho-cigano	n	Flu, abdominal pain	Brokenness ("*quebranto*"), fallen wind ("*vento-caído*")	rt, lf	H	2	2	32,568
*Acmella oleracea* (L.) R.K.Jansen	agrião	e	Flu		fl	H	1	–	32,569
*Bidens pilosa* L	picão	e	Urinary tract infection		fr	H	1	–	32,663
*Egletes viscosa* (L.) Less	macela	n	Indigestion, vomiting, abdominal pain, gastritis		fl	H	4	–	NC
*Helianthus annuus* L	girassol/sunflower	e	headache		sd	H	1	–	32,670
*Lepidaploa chalybaea* (Mart. ex DC.) H.Rob	balaio-de-velho	n*	Indigestion		lf	S	1	–	32,570
*Matricaria chamomilla* L	camomila/chamomile	e	Tranquilizer, insomnia		fl	H	2	–	NC
**Bignoniaceae**									
*Fridericia chica* (Bonpl.) L.G.Lohmann	caninana	n	Flu, kidney pain, back pain, urinary tract infection, blood cleansing		rt	L	5	–	32,675
*Handroanthus impetiginosus* (Mart. ex DC.) Mattos	pau-d'árco	n	Wounds, flu		ba	T	2	–	32,665
*Mansoa hirsuta* DC	alho-brabo	n*	Flu, sore throat		rt	L	2	–	32,677
**Bixaceae**									
*Bixa orellana* L	corante	n	Cholesterol		lf	T	1	–	32,571
**Boraginaceae**									
*Cordia rufescens* A.DC	grão-de-galo	n*	Snakebite		rt	S	1	–	32,572
*Heliotropium elongatum* (Lehm.) I.M.Johnst	crista-de-galo	n	Flu, vaginal discharge, uterine inflammation		fl, rt	H	3	–	32,573
**Bromeliaceae**									
*Ananas comosus* (L.) Merril	abacaxi/pineapple	n	Flu, cough, low immunity		fr	H	3	–	32,700
**Burseraceae**									
*Commiphora leptophloeos* (Mart.) J.B.Gillett	imburana-de-abelha, imburana-de-imbu	n	Wounds, female inflammation, uterine inflammation, ovarian inflammation, vaginal discharge, abdominal pain, kidney problems, urinary tract infection		ba	T	8	–	32,574
**Cactaceae**									
*Pereskia aculeata* Mill	ora-pro-nóbis	n	Bone pain, gastritis		lf	S	2	–	32,804
*Cereus jamacaru* DC	mandacaru	n*	Flu, kidney problems	Evil eye, protection from negative energies, envy, postpartum confinement broken ("*resguardo quebrado*")	rt, wp, fl	T	2	4	32,558
*Echinopsis oxygona* (Link & Otto) Pfeiff. & Otto	espinzinho, coroa-de-frade	n		envy, evil eye	wp	H	–	2	32,704
*Melocactus zehntneri* (Britton & Rose) Luetzelb	coroa-de-frade	n*		Evil eye, envy, protection from negative energies	wp	S	–	3	32,555
*Pilosocereus pachycladus* F.Ritter	facheiro	n*		Evil eye, envy	wp	S	–	2	32,699
*Xiquexique gounellei* (F.A.C.Weber) Lavor & Calvente	xique-xique	n*		Evil eye, envy	wp	S	–	2	32,560
**Cannabaceae**									
*Cannabis sativa* L	maconha/cannabis	e	Facial paralysis, asthma, headache, abdominal pain, epilepsy, muscle cramps		sd, lf	H	6	-	32,575
**Capparaceae**									
*Crateva tapia* L	trapiá	n	Toothache		ib	T	1	–	32,661
**Caricaceae**									
*Carica papaya* L	mamão/papaya	e	Diabetes, worms, constipation (laxative), indigestion, abdominal pain, vomiting, asthma		fr, fl, lf	S	7	–	32,557
**Cleomaceae**									
*Tarenaya aculeata* (L.) Soares Neto & Roalson	mussambê	n	Flu	Brokenness ("*quebranto*")	rt, lf	H	1	1	32,576
**Combretaceae**									
*Combretum leprosum* Mart	moita-branca	n	Flu, cough, prostate problems		ba, fl	S	3	–	32,577
*Terminalia catappa* L	castanhola	e	Kidney pain, kidney stones, gastritis		lf	T	3	–	32,578
**Convolvulaceae**									
*Operculina macrocarpa* (L.) Urb	batata-de-purga	n	Flu, cough, bronchitis		rt	L	3	–	32,656
**Crassulaceae**									
*Kalanchoe daigremontiana* Raym.-Hamet & H.Perrier	aranto	e	Cancer, urinary tract infection, diabetes, cholesterol, female inflammation		lf	H	5	–	32,701
*Kalanchoe pinnata* (Lam.) Pers	malva-santa	e	Wounds, furuncle, flu, cough, tranquilizer, inflammations, cancer, uterine inflammation, ovarian inflammation, menstrual cramps, stomachache		lf	H	11	–	32,579
**Cucurbitaceae**									
*Citrullus lanatus* (Thunb.) Matsum. & Nakai	melancia/watermelon	e	Abdominal pain, fever, urinary tract infection		sd, lf	L	3	–	32,580
*Cucurbita pepo* L	abóbora/pumpkin	e	Worms		sd	L	1	–	32,669
*Lagenaria siceraria* (Molina) Standl	cabaça	e	Wounds, furuncle		lf	L	2	–	32,660
*Luffa operculata* (L.) Cogn	cabacinha	n	Sinusitis, abortion		fr	L	2	–	32,803
*Momordica charantia* L	melão-são-caetano	e	Itchy skin, diarrhea, appendicitis, prostate problems		lf	L	4	–	32,581
**Euphorbiaceae**									
*Cnidoscolus quercifolius* Pohl	favela	n*	Wounds, inflammations, toothache		ba, lt	T	3	–	32,582
*Cnidoscolus urens* (L.) Arthur	cansanção	n	Toothache		lt	S	1	–	32,583
*Croton blanchetianus* Baill	mameleiro	n*	Flu, cough, abdominal pain		ba	S	3	–	32,584
*Croton grewioides* Baill	canilinha	n	Flu, sinusitis, cough, nasal decongestant, post-radiotherapy irritation		lf, br	S	5	–	32,585
*Croton heliotropiifolius* Kunth	velame	n	Flu, angular cheilitis ("*boqueira*")		lt, rt	S	2	–	32,586
*Euphorbia lactea* Haw	cara-de-seu-pai	e		Envy, evil eye	wp	S	–	2	32,705
*Euphorbia milii* Des Moul	coroa-de-cristo	e		Envy	wp	S	–	1	32,587
*Euphorbia tirucalli* L	cachorro-pelado, avelós	e	Cancer		lt	S	1	–	32,588
*Jatropha gossypiifolia* L	pinhão-roxo	n	Wounds, cutaneous warts	Evil eye, envy, witchcraft, brokenness ("*quebranto*"), fallen wind ("*vento-caído*"), weakness, spiritual possession, spiritual cleansing, ward off evil spirits, sleepiness	lt, lf, wp	S	2	10	32,589
**Fabaceae**									
*Amburana cearensis* (Allemão) A.C.Sm	imburana-de-cheiro	n	Healing ointmen, wounds, flu, cough, uterine inflammation, bronchitis, pneumonia, sinusitis, fever, migraine, headache, stroke, sore throat, indigestion, vomiting, abdominal pain, gallbladder pain, nasal decongestant		ba, ib, sd	T	18	–	32,806
*Anadenanthera colubrina* (Vell.) Brenan	angico	n	Flu, bronchitis, cough, expectorant, inflammations, wounds, urinary tract infection, kidney pain		ba	T	8	–	32,593
*Cenostigma bracteosum* (Tul.) Gagnon & G.P.Lewis	catingueira	n*	Abdominal pain, stomachache, constipation (laxative), gastritis, gastric ulcer, flu, cough		ba, fl	T	7	–	32,659
*Cenostigma macrophyllum* Tul	canela-de-velho	n	Bone pain, leg pain, inflamed nerve, erectile dysfunction		ba, fl	T	4	–	32,594
*Enterolobium timbouva* Mart	tamburil	n	Gastritis		ba	T	1	–	32,664
*Erythrina velutina* Willd	mulungu	n	Insomnia		ba	T	1	–	32,702
*Hymenaea velutina* Ducke	jatobá	n*	Kidney pain, back pain, bone pain, flu, abdominal pain, stomachache, vomiting, diarrhea, indigestion, inflammations		ba, sd	T	10	–	32,595
*Libidibia ferrea* (Mart. ex Tul.) L.P.Queiroz	pau-ferro	n	Flu, cough, expectorant, bronchitis, sore throat, stomachache, liver inflammation, diabetes, uterine inflammation		fr, ba	T	9	–	32,596
*Macropsychanthus grandiflorus* (Mart. ex Benth.) L.P.Queiroz & Snak	mucunã	n*	Whooping cough		lt	L	1	–	32,590
*Mimosa pudica* L	maliça	n	Urinary tract infection, sore throat, flu, itchy skin		rt	H	4	–	32,591
*Mimosa tenuiflora* (Willd.) Poir	jurema-preta	n	Wounds, inflammations, stop bleeding, dental hemorrhage, tooth inflammation, uterine inflammation, healing ointmen, bone pain	Brokenness ("*quebranto*"), fallen wind ("*vento-caído*")	ba, ib, lf	T	8	2	32,597
*Phaseolus vulgaris* L	feijão/bean	e	Anemia		sd	H	1	–	32,668
*Pityrocarpa moniliformis* (Benth.) Luckow & R.W.Jobson	rama-de-bezerro	n*	Abdominal pain, indigestion, diarrhea, flu, wounds, uterine inflammation		ba	T	6	–	32,598
*Pterodon emarginatus* Vogel	sucupira	n	Headache, flu, gastritis		sd	T	3	–	NC
*Senna occidentalis* (L.) Link	fedegoso	n	Fever	Brokenness ("*quebranto*"), fallen wind ("*vento-caído*")	lf, rt	S	1	2	32,592
*Tamarindus indica* L	tomarina	e	Constipation (laxative), diarrhea, fatty liver, cholesterol		lf, fr	T	4	–	32,599
**Lamiaceae**									
*Plectranthus amboinicus* (Lour.) Spreng	malva-do-reino	e	Flu, cough, expectorant, bronchitis, sore throat, inflammations, abdominal pain, tranquilizer, insomnia, delay aging		lf	S	10	–	32,603
*Plectranthus barbatus* Andr	boldo sete-dor	e	abdominal pain, indigestion, stomachache		lf	S	3	–	32,604
*Plectranthus ornatus* Codd	boldo	e	Indigestion, heartburn, abdominal pain, stomachache, vomiting, intestinal gas, gastritis, diarrhea, fatty liver, liver inflammation, hangover, covid		lf	H	12	–	32,693
*Mentha arvensis* L	hortelã-vique, vique	e	Flu, sore throat, nasal decongestant		lf	H	3	–	32,600
*Mentha* × *villosa* Huds	hortelã, hortelã-pimenta	e	Flu, sore throat, abdominal pain, bronchitis, sinusitis, cough, earache, fatty liver, fever, colic in babies ("*espremedeira*"), headache, hypertension, tranquilizer, vomiting, nasal decongestant, kidney problems, indigestion		lf	H	17	–	32,601
*Ocimum basilicum* L	manjericão/basil	e	Earache, toothache, sinusitis, headache, expectorant, fever, cough, flu, nasal decongestant, tranquilizer, itchy skin	Witchcraft, spiritual cleansing	lf	S	11	2	32,602
*Rosmarinus officinalis* L	alecrim/rosemary	e	Flu, headache, nasal decongestant, insomnia, tranquilizer, hypertension	Protection from negative energies, spiritual cleansing, evil eye	br	S	6	3	32,605
**Lauraceae**									
*Cinnamomum verum* J.Presl	canela/cinnamon	e	Heartburn	Ward off evil spirits, spiritual cleansing, internal agitation	ba	T	1	3	NC
*Persea americana* Mill	abacate/avocado	e	Kidney pain, kidney stones, urinary incontinence		sd	T	3	–	32,800
**Loranthaceae**									
*Struthanthus flexicaulis* (Mart.) Mart	erva-de-passarinho	n	Kidney pain		br	H	1	–	32,558
**Lythraceae**									
*Punica granatum* L	romã/pomegranate	e	Sore throat, hoarseness, cough, flu, indigestion	Attract prosperity	fr, sd	T	5	1	32,606
**Malpighiaceae**									
*Malpighia emarginata* DC	acerola	e	Flu, low immunity		fr	T	2	–	32,607
**Malvaceae**									
*Abelmoschus esculentus* (L.) Moench	quiabo/okra	e	Anemia		fr	S	1	–	32,674
*Pseudobombax marginatum* (A.St.-Hil., Juss. & Cambess.) A.Robyns	barriguda	n	Prostate problems, erectile dysfunction		ba	T	2	–	32,801
*Gossypium hirsutum* L	algodão/cotton	e	Wounds, uterine inflammation, ovarian inflammation, inflammations, flu, cough, sinusitis, uterine cleansing, diarrhea, blood cleansing, sore throat, fever, female inflammation		lf, fl, sd	S	13	–	32,608
*Hibiscus sabdariffa* L	vinagueira	e	Weight loss, anemia, low immunity		lf, fr	S	3	–	32,673
*Luehea divaricata* Mart	açoita-cavalo	n	Back pain, urinary incontinence		ba	T	2	–	32,657
**Meliaceae**									
*Azadirachta indica* A.Juss	nim	e	Back pain		lf	T	1	–	32,609
**Moraceae**									
*Morus nigra* L	amora	e	Cholesterol, cancer, menopausal hot flashes		lf	S	3	–	32,610
**Musaceae**									
*Musa paradisiaca* L	banana	e	Bone pain, tranquilizer, flu		fr, fl	H	3	–	32,658
**Myristicaceae**									
*Myristica fragrans* Houtt	noz-moscada/nutmeg	e	Stomachache, headache, indigestion, dizziness		sd	T	4	–	NC
**Myrtaceae**									
*Eucalyptus globulus* Labill	eucalipto/eucalyptus	e	Fever, flu, sinusitis, tranquilizer, nasal decongestant		lf	T	5	–	32,611
*Psidium guajava* L	goiaba/guava	e	Diabetes, diarrhea, abdominal pain, colic in babies ("*espremedeira*"), indigestion, anemia, urinary tract infection, kidney pain, liver inflammation, hair loss		lf, fr	T	10	–	32,612
*Syzygium aromaticum* (L.) Merr. & L.M.Perry	cravo	e	Sinusitis, sore throat, cough		fl	T	3	–	NC
**Nyctaginaceae**									
*Boerhavia coccinea* Mill	pega-pinto	e	Urinary tract infection		rt	H	1	–	32,613
**Passifloraceae**									
*Passiflora cincinnata* Mast	maracujá-do-mato	n	Tranquilizer, insomnia, hypertension, mental disorders		fr, lf	L	4	–	32,614
*Passiflora edulis* Sims	maracujá-peroba	n	Insomnia, tranquilizer		fr, lf	L	2	–	32,615
**Pedaliaceae**									
*Sesamum indicum* L	gergelim/sesame	e	Wounds, erysipelas, bone pain, delay aging, burn, inflammations		sd	H	6	-	32,616
**Phyllanthaceae**									
*Phyllanthus amarus* Schumach. & Thonn	quebra-pedra/stonebreaker	n	Kidney stones, kidney pain		rt, lf	H	2	–	32,617
**Piperaceae**									
*Piper tuberculatum* Jacq	pimenta-de-macaco	n	Back pain		fl	S	1	–	32,667
**Plantaginaceae**									
*Scoparia dulcis* L	vassourinha	n	Chickenpox, flu	Brokenness ("*quebranto*"), sleepiness	rt, br	H	2	2	32,618
**Poaceae**									
*Cymbopogon citratus* (DC.) Stapf	capim-santo	e	Tranquilizer, insomnia, stress, hypertension, vomiting, abdominal pain, hair loss, fatty liver, flu		lf	H	9	-	32,619
*Saccharum officinarum* L	cana-de-açúcar/sugarcane	e	Hypertension		lf	H	1	-	32,692
*Zea mays* L	milho/corn	e	Urinary tract infection		st	H	1	–	32,620
**Rhamnaceae**									
*Sarcomphalus joazeiro* (Mart.) Hauenschild	juazeiro	n	Teeth cleaning, head wound, asthma		ba, ib	T	3	–	32,621
**Rubiaceae**									
*Morinda citrifolia* L	noni	e	Cancer		fr	T	1	–	32,662
**Rutaceae**									
*Citrus limon* (L.) Osbeck	limão/lemon	e	Flu, cough, sinusitis, weight loss, cholesterol, triglyceride, gastritis, heartburn, intestinal gas, indigestion, anemia, abdominal pain, diarrhea, fatty liver, ringworm, low immunity		fr, lf	T	16	–	32,622
*Citrus sinensis* (L.) Osbeck	laranja/orange	e	Flu, migraine, abdominal pain, indigestion, insomnia, tranquilizer, fever, low immunity, diarrhea, vomiting, heartburn, labyrinthitis, pregnancy nausea		fr, lf, sd	T	13	–	32,623
*Ruta graveolens* L	arruda/rue	e	Fever, vomiting, headache, abdominal pain, eye irritation, uterine pain after childbirth ("*mãe-do-corpo*"), earache, toothache, menstrual cramps, indigestion, migraine, lice, bone pain, stroke, sore throat, tranquilizer, stomachache	Envy, evil eye, brokenness ("*quebranto*"), fallen wind ("*vento-caído*"), protection from negative energies, bad luck, bad memories, ward off evil spirits, spiritual cleansing, internal sadness, low fontanelle ("*moleira-baixa*"), witchcraft	lf, ep, br	H	17	12	32,624
**Solanaceae**									
*Capsicum frutescens* L	pimenta-malagueta	e	Furuncle, wounds	Envy	lf, wp	S	2	1	32,625
*Solanum lycopersicum* L	tomate/tomato	e	Wounds		fr	S	1	–	32,626
*Solanum tuberosum* L	batatinha	e	Gastritis		tb	S	1	–	NC
**Verbenaceae**									
*Lippia alba* (Mill.) N.E.Br. ex Britton & P.Wilson	erva-cidreira	n	Tranquilizer, insomnia, stress, migraine, headache, hypertension, fever, appetite stimulant, intestinal gas, diarrhea, flu, hangover, indigestion, constipation (laxative), edema (poor circulation), vomiting, abdominal pain, heartburn		lf	S	18	–	32,627
**Violaceae**									
*Pombalia calceolaria* (L.) Paula-Souza	papaconha	n	Flu, bronchitis, cough, expectorant, sore throat, teething		rt	H	6	-	32,628
**Ximeniaceae**									
*Ximenia americana* L	ameixa	n	Wounds, healing ointmen, female inflammation, uterine inflammation, ovarian inflammation, inflammations, flu, blood cleansing, kidney pain, prostate problems, back pain, urinary tract infection		ba, ib	T	12	-	32,696
**Zingiberaceae**									
*Curcuma longa* L	açafrão	e	Sore throat, flu		rz	H	2	–	32,629
*Zingiber officinale* Roscoe	gengibre/ginger	e	Weight loss, sore throat, cough, flu, hoarseness, delay aging, fatty liver		rz	H	7	–	32,630

*O*: origin (*e*: exotic to Brazil; *n*: native to Brazil; n*: native and endemic to Brazil); *PU*: part used (*lf*: leaf; *br*: branch; *bb*: bulb; *ba*: bark; *ib*: inner bark; *fr*: fruit; *sd*: seed; *lt*: latex; *wp*: whole plant; *rt*: root; *fl*: flower; *st*: stigma; *rz*: rhizome; *tb*: tuber); *Hb*: habit (*H*: herb; *S*: shrub; *T*: tree; *L*: liana; *PT*: palm tree); *Vph*: versatility in therapeutic targets with physical causes; *Vsp*: versatility in therapeutic targets with spiritual causes; *TEPB*: Graziela Barroso Herbarium (voucher); *NC*: not collected

Although the group of exotic species was greater than that of native species, the difference in proportions was not significant (χ^2^ = 1.51, *p* = 0.21). Furthermore, the participants made use of certain plant resources to treat both physical and spiritual illnesses. However, not every plant used to treat physical health problems was used to treat spiritual problems and vice versa.

The Poisson GLMs showed a positive relationship between the number of exotic species (dependent variable) and the number of native species (independent variable) in the therapeutic targets, with statistical significance (*p* < 0.05) in both groups (physical and spiritual). However, when the influence of the amount of native plants on the presence or absence of exotic species in the therapeutic targets was investigated (Binomial GLMs), no significant relationships (*p* > 0.05) were identified in any of the groups, as shown in Table 2.

**Table 2:** Results of Poisson and Binomial GLMs in therapeutic targets with physical and spiritual causes in Morrinhos

Therapeutic targets	GLM	Estimated value	Standard error	*z* value	*p* value
Physical	Poisson	0.098327	0.006628	14.84	< 0.001***
	Binomial	0.1595	0.1266	1.260	0.208
Spiritual	Poisson	0.23871	0.06033	3.957	< 0.001***
	Binomial	0.1765	0.3062	0.576	0.564

Significance levels: *p* > 0.05; ***p ≤ 0.001

**Fig. 2:**
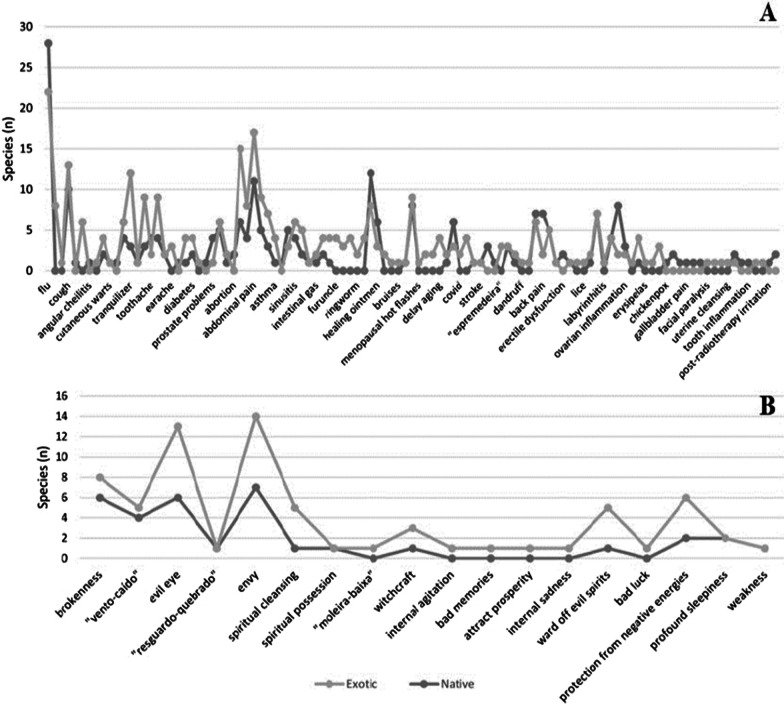
Distribution of exotic and native species in therapeutic targets with physical (**A**) and spiritual (**B**) causes in Morrinhos

We observed a significant overlap between native and exotic species in targets with physical and spiritual causes. Notably, targets treated with a variety of exotic plants were also treated with many native species, contrary to our expectations (Fig. 2). Furthermore, the presence of exotic species in these therapeutic targets was not related to the presence of native species (*p* > 0.05). Based on these results and the data collected, the diversification hypothesis was not able to consistently explain the role of exotic species in the therapeutic groups evaluated in the community, at least in the current context.

As for the versatility of the species, no significant differences were found between native and exotic species regarding indications for therapeutic targets with physical (*p* > 0.05) or spiritual (*p* > 0.05) causes. Therefore, therapeutic versatility between native and exotic resources was similar in both groups, as shown in Table 3. It is noteworthy that, despite the absence of significant differences in versatility, the mean of exotic species in targets of physical nature was higher. Among the 10 most versatile species, only 3 were native. The exotic species *Dysphania ambrosioides* (L.) Mosyakin & Clemants (Amaranthaceae) (24), *Aloe vera* (L.) Burm.f. (Asparagaceae) (20), *Ruta graveolens* L. (Rutaceae) (17), and *Mentha* × *villosa* Huds. (Lamiaceae) (17) stood out with a greater number of indications for physical therapeutic targets. Among the native species, *Amburana cearensis* (Allemão) A.C.Sm. (Fabaceae) and *Lippia alba* (Mill.) N.E.Br. ex Britton & P.Wilson (Verbenaceae) had 18 indications, while *Astronium urundeuva* (M. Allemão) Engl. (Anacardiaceae) stood out with 15. In the group of spiritual targets, there was a smaller number of species and therapeutic indications. The most versatile included *R. graveolens* (12 therapeutic indications), *Jatropha gossypiifolia* L. (Euphorbiaceae) (10), *Allium sativum* L. (Amaryllidaceae) (4) and *Cereus jamacaru* DC. (Cactaceae) (4).

**Table 3:** Versatility of native and exotic plants in therapeutic targets with physical and spiritual causes in Morrinhos

Versatility	O	Me	Md	SD	V	Q1	Q3	Mann–Whitney
Physical therapeutic targets	e	5.1	3	5.21453	27.19135	1	6	W = 1967, p = 0.4401
	n	4.23	3	4.18218	17.49058	1.75	5	
Spiritual therapeutic targets	e	2.78	2	2.83328	8.02747	1	3	W = 86 p = 0.9358
	n	2.75	2	2.41680	5.84091	2	2.25	

*O*: origin (*e*: exotic to Brazil; *n*: native to Brazil); *Me*: mean; *Md*: median; *SD*: standard deviation; *V*: variance; *Q1*: 1st quartile; *Q3*: 3rd quartile

With regard to the set of redundant illnesses with physical causes, exotic species were prioritized over native ones (χ^2^ = 79.15, *p* < 0.001). A different result was found in illnesses with spiritual causes: native species were prioritized (χ^2^ = 4.09, *p* = 0.043). Thus, in this perspective, there was a differentiation in the behavior of the medical system with respect to the influence of exotic and native resources on their dynamics of use, depending on the type of treatment.

## Discussion

### Diversification hypothesis in therapeutic targets with physical and spiritual causes

In the current context of Morrinhos, the diversification hypothesis [4] does not seem to explain the role of exotic species in the local medical system. At this point, it is also important to question the definition of the biogeographic origin of the species, since the very classification of a species as “exotic” can be influenced by political and cultural factors [42]. Therefore, in future studies on the interaction between people and plants, especially when evaluating the influence of the origin and role of plants in LMS, it is important to take into account not only etic criteria determined by the interpretation of the researchers, but also the emic perception of the people involved, how they understand the introduction of the species in their therapeutic demands, and how they perceive a resource as “exotic” and “native” in their environment, without the biases of the associated political, cultural and environmental circumstances. As an example, in French Guiana, a recent study explored the emic view of quilombolas in relation to invasive exotic tree species and highlighted that these peoples often consider these plants as native to their socio-ecological systems, making etic definitions of little significance for them [43].

A systematic analysis of the diversification hypothesis in Brazilian biomes at different levels (national, regional and local) conducted by Medeiros et al. [44] revealed disparities between the main Brazilian ecosystems, from small differences to considerable overlap between native and exotic plants (*p* > 0.05), suggesting weaknesses in this hypothesis depending on the scope of the analysis. In Ecuador, introduced plants were more likely to be used to treat diseases with fewer native plant treatment options and to fill therapeutic gaps, lending support to the central idea of the diversification hypothesis [12]. However, cultural and environmental differences need to be taken into consideration when we compare these results [8], since the very understanding of what constitutes an illness can vary from place to place [11].

In this sense, in agreement with the literature, the incorporation of exotic plants aimed at targets already well covered by native species may represent an adaptive strategy of local communities to enhance the resilience of their medical systems, increasing the utilitarian redundancy and, consequently, the flexibility of alternatives, in addition to serving as a buffering mechanism against possible environmental disturbances that may compromise one or more species [15, 19]. The ability of the system to respond to disturbances is thus enhanced, ensuring the continuity of healing practices, whether physical or spiritual, by human groups. Furthermore, it is noted that human groups tend to incorporate plants into their medical systems not to favor redundancy in a large number of therapeutic targets, but rather in a particular set of targets locally considered the most important, thus suggesting that the inclusion of plants into LMS, regardless of the biogeographic origin of the resources, follows the logic of protecting specific therapeutic targets.

In line with this idea, studies carried out in rural communities in the semiarid region of Ceará found that the more redundant therapeutic targets were the most common and least severe, since the participants concentrated their efforts in gaining knowledge in events that were more likely to occur, promoting the resilience of the system [45, 46]. We understand that investing in strategies to increase redundancy in targets that rarely affect people is costly and unsafe, as the frequency of disease onset in the environment can be so low that the memorization of resources becomes difficult, compromising their use when necessary and the persistence of this information in time and space.

Experiments on adaptive memory and the evolution of the human mind in the context of medicinal plants found that people tend to remember more easily plants associated with common and less serious health problems [47]. This mnemonic capacity associated with these diseases can be explained by the high frequency with which they occur and by previous personal experiences, which facilitates the process of remembering therapeutic information [47]. Following this reasoning, we may look into the possibility that the diversity of plants in the most redundant therapeutic targets may have been favored by the recall of several species that treat the most common diseases of physical and spiritual nature, regardless of their origin, while non-overlapping and/or little redundant targets were affected by the lower retention of data in memory, possibly due to less frequent indications in the community.

### Versatility in therapeutic targets with physical and spiritual causes

The therapeutic versatility of exotic and native resources was similar in the two groups of illnesses studied, contrary to what was initially proposed in the definition of the versatility hypothesis by Bennet and Prance [10]. Our findings differ from those of Hart et al. [12] in the Ecuadorian medicinal flora, in which greater therapeutic versatility was found for exotic species. Hart et al. [12] attributed this finding to the fact that the wide geographical distribution of exotic species, which were generally cosmopolitan, increased the chances of use, expanding discoveries and therapeutic possibilities, leading to the selection of exotic species based on their versatility. However, this same premise was not validated in other scenarios with drier conditions. In the semiarid region of Pernambuco, Alencar et al. [6] found that native plants were more versatile in general uses, with no significant differences between native and exotic plants in terms of therapeutic versatility. Likewise, no significant differences were found between native and exotic species in the Cerrado of Minas Gerais [8]. And in the present study, no differences were found either. Thus, the selection of exotic species in these systems, although geographically and culturally different, is not supported by the variety of therapeutic indications.

In these circumstances, we may consider the possibility that the differences observed in the studies may be associated with seasonality patterns in ecosystems and the way people have evolutionarily incorporated plants in the construction of their medical systems over time. In Ecuadorian ecosystems, seasonality is less pronounced [12] and does not play an important role as in Caatinga and Cerrado. The annual availability of plant resources is not a limitation for plant use patterns in the same way as in drier environments, where the temporary availability of many plants leads to greater attention and use of specific resources, such as woody species and their perennial parts, such as roots and bark [4, 48]. In addition, in past times, droughts in semiarid regions limited the access to water, posing difficulties to the management of several plant species, especially exotic plants in these areas.

Based on the assumptions presented above, the resources most likely to be found and used throughout the year in drier ecosystems, such as woody species and their permanent parts, have particularities that favor their frequent use, promoting experimentation and maximization of therapeutic benefits in local populations [49]. This may lead to the valorization of certain native species over time and consequent discovery of a variety of therapeutic functions, contributing to their versatility in relation to ephemeral species, including many exotic herbaceous species. This probably does not apply to Ecuadorian ecosystems. Thus, we believe that these factors have influenced the discovery of therapeutic properties of native plants and, thus, their versatility in drier environments. However, the advent of improvements in access to water for various purposes, including the cultivation and facilitation of the use of many exotic species, is a factor to be taken into account. Such improvements and issues related to seasonality together may have contributed to the variety of therapeutic indications and, thus, the similarity observed in terms of versatility between native and exotic plants in the present study. In this study, for example, we observed that most of the exotic plants mentioned by the participants were herbaceous species generally with a restricted distribution in the environment because of climatic seasonality or kept in backyards under human management and care, while native species consisted mostly of arboreal plants.

Plants with a wide range of medicinal applications have particularities that favor their selection by human groups [6]. In the Morrinhos community analyzed in this study, rue (*R. graveolens*), a plant native to the Mediterranean area and Southwest Asia [50], stood out with high versatility in therapeutic targets with physical and spiritual causes. Several ethnobotanical works have reported the use of this species for physical and spiritual purposes [21, 30, 31]. These results demonstrate that the same plant resources can satisfy targets of different natures in a therapeutic continuum that involves physical and magical aspects. The experimentation and inclusion of these plants may follow a similar dynamic in both contexts, with their therapeutic functions intertwining in the promotion of personal well-being.

### Utilitarian redundancy and prioritization in therapeutic targets with physical and spiritual causes

Two scenarios were found regarding the prioritization of resources in the evaluated targets: exotic plants were prioritized in diseases of physical nature and native plants were prioritized in those of spiritual nature. To explain these differences, we propose two main influencing factors, according to the context analyzed: 1) the current ease of access to exotic plants, which are usually grown in home gardens and backyards, favoring their frequent use to treat physical diseases; 2) and the more intense connection between historical-cultural factors and spiritual problems, creating a certain resistance to the prioritization of redundant exotic resources, at least in the short term; people prefer to use native plants that have played a magical-religious role over generations.

In recent decades, public policies have affected the access to water in the Brazilian semiarid region, including the implementation of water supply systems through artesian wells and the adoption of cisterns and other reservoirs to collect rainwater, and this has contributed enormously to the reconfiguration and diversification of the water use pattern [51]. These measures have not only mitigated the effects of droughts, but also enabled and/or facilitated the maintenance of backyards and the cultivation of plants for subsistence [52], including medicinal species and, among them, many exotic plants whose cultivation would not otherwise be feasible. Thus, several exotic medicinal species are grown in backyards in the Northeastern semiarid region of Brazil [1, 53, 54], and this proximity with these plants may influence the choice of medicinal elements. In decision making, the perception of availability of plants can be influenced by their accessibility [55], since an abundant species can be perceived as unavailable if it occurs far from homes [56].

Environmental degradation caused by human activities is a critical factor that reduces the availability of forests for plant collection, implying greater time and effort required to obtain native plants [57]. At the same time, and in line with the ecological appearance hypothesis [4], local exotic plants in the current scenario, especially those grown and kept in domestic backyards at the service of people, in this case, consumers, end up becoming the most “apparent” in the community, that is, the most likely of being found and collected [56]. The reduction and distance of native forests, therefore, may have influenced the prioritization and consumption of exotic resources. This complex dynamics among access to water, plant use, and environmental changes can be fundamental to understand the choices of resources to treat physical diseases in semiarid communities such as Morrinhos.

Due to their multifunctionality, local backyards can work as therapeutic reservoirs and their medicinal resources tend to be prioritized, a trend that may also be present in other communities with similar conditions. In turn, this prioritization can bring benefits to the system from an adaptive and also environmental point of view, since the use-pressure concentrated on exotic resources somehow minimizes the impact on native species with redundant indications, ultimately contributing to their preservation [18, 19], besides maintaining local functions, and favoring the resilience of the system [14].

On the other hand, several exotic plants are often portrayed as species with high invasive potential, constituting the second largest threat to biodiversity [43] and bringing a negative impact on the conservation of native species [58]. However, in areas of dry forests, such as Caatinga, many exotic species only persist in favorable environments, such as backyards, due to human intervention, through management and care in their cultivation [20, 40], because most of them are unable to propagate and survive in areas of dry native forest, thus reducing the risks of invasion and the subsequent ecological impacts [59].

In the light of biocultural adaptation, Gama et al. [20] report that in certain environmental contexts there is a tendency for introduced plants to offer advantages over native ones due to the perception of greater therapeutic efficacy, palatability and availability. Yet the authors point out that, despite the advantages, these species only stand out in LMS when the cost–benefit ratio is favorable. Therefore, extrapolating this idea to our data, we can assume that many exotic species used to treat diseases of physical nature are not only the “most accessible” under current conditions, but also have a favorable cost–benefit ratio. These results show the dynamicity of culture and how socio-environmental variations can influence use patterns and prioritization within the system.

With regard to the prioritization of native plants for spiritual purposes, a plausible explanation may be the strong cultural attachment to these plants. Social and cultural factors can influence the selection process and use patterns [4]. Among plants with magical-religious use, we can infer that some native plants play the role of key cultural species in this context, whose existence in the community and intrinsic spiritual value are fundamental to the stability of spiritual therapeutic practices over time, given their symbolic and ritualistic role [60] and cultural prestige in the collective opinion that is difficult to replace [61]. Based on the concept of cultural keystone species, Tareau et al. [62] developed the definition of “spiritual keystone species” in reference to this symbolic, magical and spiritual centrality of certain species in magico-religious practices. In this study based on Neotropical cosmovisions, the authors highlighted the Amazonian tree *Ceiba pentandra* (L.) Gaertn (Malvaceae) as a significant “spiritual keystone species” in indigenous and Afro-descendant communities across America [62]. So, species of the native flora can play a very relevant role in the local cultural identity [61], especially when it comes to spiritual matters.

In line with the above, Bussmann and Sharon [63] found that most of the plants used in the treatment of magical/ritual ailments in Northern Peru come from mountainous forests of the Andes, especially those found in the vicinity of sacred sites, as they are considered more powerful and energetic and tend, thus, to be the most used in the Andean culture. Similarly, local communities in India believe that local trees play a central role in magical-religious processes, so that they maintain the knowledge and a close relationship with these native resources [64].

In a rural region of the Brazilian Amazon, Kawa [30] observed that species native to the Brazilian territory, such as *J. gossypiifolia* (“pinhão-roxo”), were the magical-religious plants most cultivated in the backyards for spiritual ailments. In rural backyards of South-Central Piauí, native species were the most cultivated for this purpose, especially *J. gossypiifolia* and *Melocactus zehntneri* (Britton & Rose) Luetzelb. (Cactaceae) [31]. The deliberate management of these species seems to be related to the preference of the maintainers for these plants, the greater confidence in their magical-religious efficiency, and the cultural link with these resources compared to other species, such as many exotic species used for similar purposes. In a study with prayers in Maranhão, Rabelo, Araújo and Almeida Júnior [65] did not find exclusivity in the use of magical-religious plants, but perceived a clear preference for two native plants (*J. gossypiifolia* and *Scoparia dulcis* L.—Plantaginaceae) due to the perception of their mystical efficiency and because of the greater cultural affinity with them.

Based on the above, the preference for certain magical-religious plants in the treatment of spiritual ailments permeates symbolic phenomena and is intrinsically linked to the cultural formation of each human group, as well as to their cosmovisions, identities, and empirical perceptions. In the context of this immateriality, it is believed that the cultural proximity of “sertanejo” human groups (inhabitants of the Northeastern semiarid region), such as the residents of Morrinhos, with native plants generates a more intense socio-cultural connection with these resources. Thus, the relationship between plants, the cultural context, and the identity of each group can be understood as playing a relevant role in the perception and appreciation of magical-religious resources in the context of ailments with spiritual causes.

Within the scope of this discussion, it is also important to highlight that some publications [66, 67] suggest that the use of plants in certain spiritual treatments, such as in rituals that involve direct contact with the patient’s body, can be based not only on magical properties attributed to these plants, but also on their active ingredients. In this sense, another challenging task pointed by Pagani, Santos and Rodrigues [68] is establishing a correspondence between the emic terms of traditional medicine and the symptoms or diseases known by conventional medicine. The authors demonstrated that some Culture-Bound Syndromes (CBS) resemble physical illnesses more than others. Therefore, in CBS in which there is a strong connection with the local belief system and cosmovision, it can be challenging to accurately discern the boundaries between magical and physical or between scientific and spiritual [21].

It is noteworthy that the priority given to native plants for magical-religious purposes may eventually create or intensify risks to these species. The family Cactaceae has a considerable number of species endemic to the Northeastern semiarid region of Brazil, such as *Melocactus* spp., among others, which are used in various ways in the “sertaneja” culture, including for magical-religious purposes [31, 69–71], as observed in the present work. Thus, the use-pressure on these resources can pose or aggravate threats to their diversity. This is especially true in the case of certain cacti of the genus *Melocactus* Link & Otto which occur mainly in Bahia and already appear as Critically Endangered (CR) or in other statuses of concern in the National List of Endangered Species [72]. The main threat to populations of these succulent plants comes from the extraction for commercialization in open markets and highways, intended for various forms of use [73]. Cultural factors and the additional uses of these plants for spiritual ends may affect the trade dynamics and extraction may be intensified. This reinforces the need for sustainable actions to preserve these plants and highlights that the involvement of local communities and the respect for their traditional knowledge are essential to ensure that magical-religious processes coexist harmoniously with environmental preservation.

## Conclusions

In this study, the diversification and versatility hypotheses were not able to explain the role of exotic plants in illnesses with physical and spiritual causes. This indicates that the ideas in these hypotheses may not be sustained in communities of the Caatinga, such as Morrinhos, due to specific environmental and sociocultural factors of these environments. Regarding the patterns of use of plant resources in the treatment of physical and spiritual illnesses, the system had similar and distinct patterns in the exotic and native plants, depending on the perspective evaluated. This finding shows the importance to avoid limiting studies on medicinal plants to therapeutic indications involving physical illnesses, neglecting the magical-religious uses that can play a significant role in LMS, or treating them as a single therapeutic group, because although the symptoms may overlap, the causes are perceived in different ways.

The expansion of investigations on the relationship between peoples and plant symbolism is sorely needed. Future studies should consider the diversity of cultures, understanding the patterns that govern them and recognizing the importance of these medical systems. It is essential to explore the dynamics in groups with different empirical perceptions, such as indigenous peoples, prayers, quilombolas, “terreiro” peoples, gypsy groups, rural workers, riverside dwellers, among others, taking into account divergent socio-cultural, economic, environmental and contextual issues in order to better assimilate the behavior of these therapeutic targets with different causes. Furthermore, it is important to deepen the understanding of physical and spiritual boundaries in these contexts. These approaches will undoubtedly broaden the debate on the role of native and exotic plants in health processes, as well as on how the biogeographic origin of the plants can influence their use in folk medicine for illnesses with physical and spiritual causes.

## Data Availability

All data generated or analyzed during this study are included in this published article.

## Abbreviations

LMS: Local medical system
DH: Diversification hypothesis
GLM: Generalized linear model
URM: Utilitarian redundancy model
FHP: Family Health Program
PI: Piauí
CAAE: *Certificate* Of Presentation for *Ethical Appreciation*
ICF: Informed Consent Form
SisGen: National System of Genetic Resource Management and Associated Traditional Knowledge
SISBIO: Biodiversity Information and Authorization System
UFPI: Federal University of Piauí
TEPB: Graziela Barroso Herbarium
APG: Angiosperm Phylogeny Group
spp.: Species
χ^2^: Chi-square test statistic
CR: Critically Endangered
O: Origin
Me: Mean
Md: Median
SD: Standard deviation
V: Variance
Q1: 1St quartile
Q3: 3Rd quartile
e: Exotic to Brazil
n: Native to Brazil
n*: Native and endemic to Brazil
PU: Part used
lf: Leaf
br: Branch
bb: Bulb
ba: Bark
ib: Inner bark
fr: Fruit
sd: Seed
lt: Latex
wp: Whole plant
rt: Root
fl: Flower
st: Stigma
rz: Rhizome
tb: Tuber
Hb: Habit
H: Herb
S: Shrub
T: Tree
L: Liana
PT: Palm tree
Vph: Versatility in therapeutic targets with physical causes
Vsp: Versatility in therapeutic targets with spiritual causes
NC: Not collected
